# DELIRIUM SEVERITY AND COGNITIVE OUTCOMES

**DOI:** 10.1093/geroni/igz038.3356

**Published:** 2019-11-08

**Authors:** Sarah A Welch, E Wesley Ely, Jin H Han

**Affiliations:** 1 Vanderbilt University Medical Center, Nashville, Tennessee, United States; 2 Department of Emergency Medicine, Department of Medicine, Vanderbilt University Medical Center, Nashville, Tennessee, United States

## Abstract

Delirium is heterogeneous and can vary by severity. The impact of its severity is unclear. This prospective cohort study enrolled emergency department (ED) patients who were > 65 years old and admitted to the hospital. Delirium severity was determined by the Confusion Assessment Method for the Intensive Care Unit Severity (CAM-ICU-S) Scale measured at enrollment. This scale ranges from 0 (no symptoms) to 7 (most severe). Premorbid and 6-month cognition were determined using the Informant Questionnaire on Cognitive Decline in the Elderly (IQCODE) which ranges from 1 to 5 (severe cognitive impairment). Multiple linear regression was performed to determine if delirium severity was associated with 6-month function and cognition adjusted for pre-illness IQCODE, baseline functional status, comorbidity burden, severity of illness, and central nervous system diagnosis. Two-factor interactions were incorporated to determine if pre-illness cognition modified the relationship between delirium severity as measured by the CAM-ICU-S and 6-month cognition. A total of 228 older patients were enrolled in the ED and of these, 105 were delirious. Median (interquartile range) CAM-ICU-S scores was 2 (0, 5). In patients with intact pre-illness cognition, a point increase in the CAM-ICU-S significantly increased the 6-month IQCODE by 0.06 (95%CI: 0.01 to 0.12) points. In patients with impaired pre-illness cognition, there was no significant association between the CAM-ICU-S and 6-month IQCODE. Thus delirium severity is associated with poorer 6-month cognition, but this association is more prominent in those with intact pre-illness cognition.

